# Multifaceted Support for a New Medical School in Nepal Devoted to Rural Health by a Canadian Faculty of Medicine and Dentistry

**DOI:** 10.5539/gjhs.v4n6p109

**Published:** 2012-09-10

**Authors:** Kim Solez, Arjun Karki, Sabita Rana, Holli Bjerland, Bibiana Cujec, Stephen Aaron, Don Morrish, MaryAnn Walker, Manjula Gowrishankar, Fiona Bamforth, Lalith Satkunam, Naomi Glick, Tom Stevenson, Shelley Ross, Sanjaya Dhakal, Dominic Allain, Jill Konkin, David Zakus, Darren Nichols

**Affiliations:** 1 Faculty of Medicine and Dentistry, University of Alberta, Edmonton, Alberta, Canada; 2 Patan Academy of Health Sciences, Kathmandu, Nepal

**Keywords:** urban rural disparity, global health, Nepal, Canada, competency based assessment, peace-making through health equity, medical education, University of Alberta

## Abstract

Nepal and Alberta are literally a world apart. Yet they share a common problem of restricted access to health services in remote and rural areas. In Nepal, urban-rural disparities were one of the main issues in the recent civil war, which ended in 2006. In response to the need for improved health equity in Nepal a dedicated group of Nepali physicians began planning the Patan Academy of Health Sciences (PAHS), a new health sciences university dedicated to the education of rural health providers in the early 2000s.

Beginning with a medical school the Patan Academy of Health Sciences uses international help to plan, deliver and assess its curriculum. PAHS developed an International Advisory Board (IAB) attracting international help using a model of broad, intentional recruitment and then on individuals’ natural attraction to a clear mission of peace-making through health equity. Such a model provides for flexible recruitment of globally diverse experts, though it risks a lack of coordination. Until recently, the PAHS IAB has not enjoyed significant or formal support from any single international institution.

However, an increasing number of the international consultants recruited by PAHS to its International Advisory Board are from the University of Alberta in Edmonton, Alberta, Canada (UAlberta). The number of UAlberta Faculty of Medicine and Dentistry members involved in the project has risen to fifteen, providing a critical mass for a coordinated effort to leverage institutional support for this partnership. This paper describes the organic growth of the UAlberta group supporting PAHS, and the ways in which it supports a sister institution in a developing nation.

## 1. Introduction

Sharing knowledge and experiences for the betterment of medical education in developing countries is one of the key benefits of global health education endeavors. In this paper we describe a program that is being developed as a joint venture between a group at a Canadian university and their colleagues at a new health sciences university, the Patan Academy of Health Sciences (PAHS) in Nepal. The intention of this collaboration is to build educational programs that increase health services for rural populations in Nepal. This paper focuses on the rapid, yet effective evolution of a PAHS support group at the University of Alberta (UAlberta) and how it has initiated a multifaceted collaboration at PAHS.

Located in Kathmandu, The Patan Academy of Health Sciences admitted the first students to its medical school in 2010. Unique to Nepal, PAHS recruits and educates students to provide rural health care – a key step in reducing the rural-urban disparity that helped ignite a decade-long civil war in 1996. More than just a medical school, PAHS is a gateway to Nepal’s most needy communities, where global innovations in health education are taking root. While the Academy has started with a medical school, it is dedicated to the education of not only rural physicians, but also nurses, midwives, public health professionals and other health care providers. Having adopted best practices from around the globe, PAHS is actively engaged in solving the health care inequity in one of the world’s poorest nations (Courneya & Dunne, 2009; Karki, Courneya, & Woollard, 2009).

The group from the University of Alberta is made up of diverse members from within the Faculty of Medicine and Dentistry who share a common interest in Nepal, the betterment of its health care system and improvement of its people’s health. This Nepal group provides multifaceted support in areas where PAHS has identified needs, from fund development, to pedagogy, to program development and research.

The UAlberta Nepal group is hoping to help PAHS become an example of innovative and sustainable medical training in the developing world, built upon a framework of competency-based medical education, community responsiveness and effective international collaboration.

**Figure 1:**
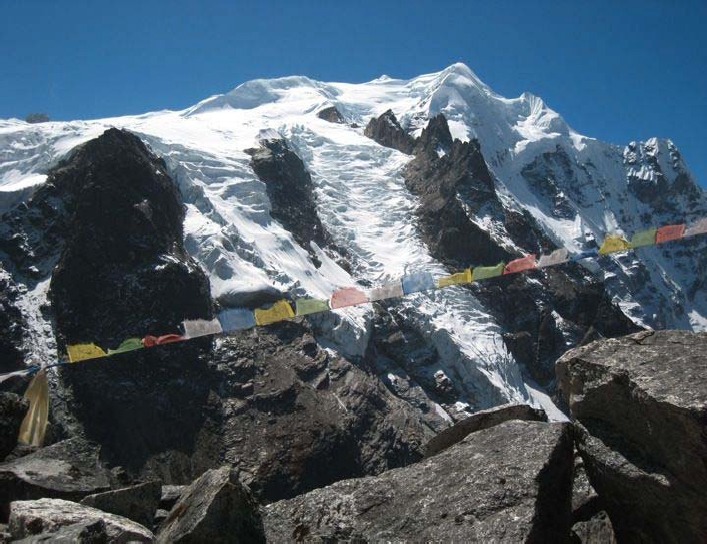
The rugged but beautiful scenary of Nepal

## 2. Background

### 2.1 Challenging Demographics

With a population of 27 million, and only 17% of its citizens urbanized, Nepal lives with a constant rural-urban disparity in health. A powerful caste system, limited possibilities for advanced education or travel, a strong economic divide, a recent decade of civil war and subsequent political instability mean the urban-rural health divide remains. While advances have been made in the last quarter century on the targets of the Millennium Development Goals, progress in bringing health equity to the Nepal’s 22 million rural residents remains slow. Average national life expectancy has risen to 66.5 years (with significant rural-urban differences), but literacy is only 33% among women. Infant mortality (46/1000) and maternal mortality (281/100,000) are in the bottom 25% worldwide (Ministry of Health and Population (MOHP) [Nepal], 2012). A robust 5 hospital beds for every 1000 population does not reflect that those beds have few nurses and few doctors to tend to them. A reported rate of 1 physician per 5000 is an average that does not reflect the profound lack of rural medical care (Source: CIA Factbook Table 1).

**Table 1:** 

	Kathmandu	Hills	Mountains	Canada
Doctors	1:850	1:30000	1:100 000	1:450
Infant Mortality^1^ (Per/1000)	33	47	99	5
Life Expectancy^2^ (World Rank)	65.5 (168th)	<65	~40	81.2 (8th)

1 *. Ministry of Health and Population (MOHP) [Nepal], New ERA, and Macro International Inc. 2007. Nepal Demographic and Health Survey 2006*.

2 *. Woollard R (2005). Feasibility Study for the Proposed Patan University of Health Sciences (PUHS), Medical School Steering Committee, Kathmandu*.

### 2.2 A Wealth of Physicians in the Wrong Places

Nepal produces around 2000 new physicians per year in Kathmandu and other urban centers. The vast majority of the students come from wealthy urban families. Upon completing medical school, many seek greener pastures for post-graduate training and /or employment outside Nepal (Shankar, 2010).

For those physicians who remain in Nepal, few practice outside the urban setting – a problem also encountered in Canada. Of those who find post-graduate education in Nepal, very few do general (family) practice. The result is an excess of under-resourced urban specialists with no unifying primary care system, and virtually no doctors for the rural majority in Nepal.

### 2.3 A Brief History of the Patan Academy of Health Sciences

At the turn of this century a dedicated core of Nepali physicians came together to create a new health sciences university, one that would help to remedy the health inequity of their nation. From the outset, the mission of the Patan Academy of Health Sciences was to sustain improvement of the health of the people of Nepal, especially those who are poor and living in rural areas, through innovation, equity, excellence and love in education, service and research. After years of national and international collaboration, and not without significant struggle, the founding faculty members succeeded. On August 20, 2007 the Government of Nepal endorsed the concept proposal and PAHS came into legal existence on February 6, 2008 with the Charter granted by the Parliament of Nepal.

PAHS is unique in its inception, its vision and its support. It is the only medical school in Nepal designed to educate and retain rural physicians. Led by its then Vice Chancellor, Dr. Arjun Karki, and his team, PAHS adopted exemplary educational practices from around the globe. Together with an International Advisory Board, the leaders of PAHS have indigenized some of the most successful admissions, curricula and assessment in medical education. Spearheaded by a curriculum that integrates students into rural communities PAHS aims to create not just doctors, but medical leaders and agents of change. To do so they employ a unique admissions process that preferentially selects for capable students from poorer rural and remote districts.

The Patan Academy of Health Sciences is housed at the Patan Hospital. Founded in 1956 by the United Missions to Nepal; its ethos of service to all patients regardless of ability to pay enshrined Patan Hospital as a top centre for healthcare in Nepal. In 2006, Patan Hospital was handed over to the government of Nepal, but the hospital’s mission remains unchanged: to provide a caring, effective and efficient hospital-based health service for any person in need who presents to the Hospital.

Today Patan Hospital is one of the largest hospitals in Nepal. Using modern equipment it provides treatment for almost 320,000 outpatients and 20,000 inpatients, including 10,000 surgeries every year. The hospital has been operating with an annual revenue of around US $3.5 million, making great use of this limited resource.

**Figure 2:**
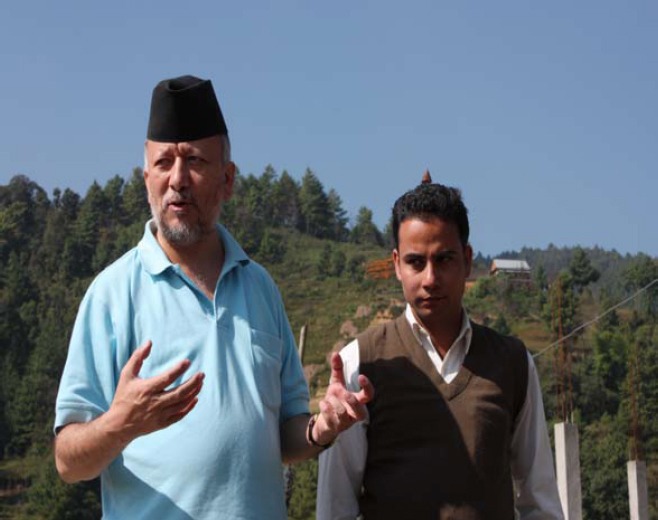
Dr. Arjun Karki in rural Nepal

PAHS provides scholarships to 60% of its students (40% of students pay only half the fee and 20% pay no tuition fee), which puts a substantial financial burden on the institution, hence the importance of making best use of limited resources.

### 2.4 Research at Patan Academy of Health Sciences

While just starting out, the research programs at the PAHS and the Patan Hospital are already punching above their weight. Several areas of expertise are already developing. Patan is the current world leader in enteric fever research, and has produced top tier publications (Arjyal A et al., 2011; Holt KE, et al., 2010; Karkey et al., 2008, 2010; Kelly et al., 2011; Koirala et al., 2012; Maskey AP, et al., 2006, 2008; Murdoch DR, et al., 2008;Pandit A, et al., 2007, 2008; Zimmerman M et al., 2008), which have changed the way in which typhoid fever is treated world-wide.

Innovations in education (Adhikari N, 2010;Butterworth, 2010;Butterworth & Hayes, 2010; Butterworth, Hayes & Neupane 2008;Butterworth, Hayes, & Zimmerman, 2011; Butterworth & Pradhan, 2010; Upadhyay, Bhandary, & Ghimire, 2011) and health policy (Allaby, 2003;Gongal et al., 2009; Gongal & Bhattarai, 2005; Gongol & Maharjan 2010; Hari, Mark, Bharati, & Khadka, 2007; Hayes, 2008) have been two other areas of publishing strength for the institution. The hospital also has been a source of telemedicine expertise for more than ten years (Graham et al., 2003).

## 3. Meeting the challenges of medical education in Nepal

Major challenges common to many medical schools in developing countries include low levels of funding, shortages of academic staff, limited faculty development and a pressing need to continue and evolve clinical service delivery.

The challenges at PAHS are similar to those of many developing world medical schools. In addition to the demands of creating competent physicians, a significant added challenge exists: students must develop identities as a rural physicians as they pursue a mixed urban-rural education (Konkin & Suddards, 2011; Jarvis-Selinger, Pratt, & Regehr, 2012).

Under the leadership of its founding Vice Chancellor, Dr. Arjun Karki, PAHS developed a very powerful tool to combat these challenges in the form of an International Advisory Board (IAB) chaired by Prof. Cliff Tabin from Harvard Medical School. The IAB enjoyed a very organic growth, with members being invited to join, and then staying on as their passion, interests and skills were found to match the needs of PAHS. At the request of PAHS faculty, the IAB members have advised on feasibility, admissions, curriculum and assessment, filled gaps in pre-clinical teaching, and provided faculty development and fund raising. Guided by their natural attraction to the mission of the PAHS, new IAB members continue to join, outpacing the normal attrition of volunteer members. Nowhere is this growth more evident than with IAB members from the University of Alberta who have had a rapid evolution in their support of the medical school.

## 4. The University of Alberta’s Nepal Group

### 4.1 The History of the UAlberta’s Connection

Alberta’s medical humanitarian involvement in Nepal goes back to 1960 and the efforts of Dr. Helen Huston (Hankins, 1993). As a matter of fact, technical assistance from the University of Calgary during the late 1970’s and early 80’s was instrumental in establishing Nepal’s first medical school, the Institute of Medicine of Tribhuvan University. The modern era began in 1998 with the cyberNephrology initiatives of Dr. Kim Solez providing electronic educational material and Internet connections to the renal units in Nepal. In 1999 the new Chair of Laboratory Medicine and Pathology at the University of Alberta, Victor Tron, suggested a broadening of approach from cyberNephrology to cyberMedicine (Solez, Hales & Katz 2005). This ultimately led to Dr. Solez joining the medical advisory board of PAHS. A chance cafeteria discussion in 2007 with educator Dr. David Cook led to the discovery that he was also helping PAHS, providing curriculum development and teaching effectiveness workshops. Following Dr. Cook’s untimely death in 2009 the number of University of Alberta faculty involved in the project rapidly grew to the present fifteen. His widow Maryann Walker has become the vibrant force behind fund raising efforts for the project in David Cook’s name.

In 2011 Dr. David Zakus came to the University of Alberta as the new director of the Office of Global Health in the Faculty of Medicine and Dentistry. His leadership has given much energy and organization to the project and has provided an academic home for it at the University of Alberta. In 2012 Dr. Dominic Allain was added as Deputy Director of the Office of Global Health.

### 4.2 The International Advisory Board

The University of Alberta faculty and staff who are actively supporting PAHS understand that they can only meet a handful of specific needs. However, the group has been able to provide support in a large number of areas. A number of faculty members sit on the International Advisory Board (IAB) and provide advice and consultation at the Annual Consultative Meeting. The IAB is very active providing volunteer international faculty and external review (evaluation and assessment) of PAHS activities.

**Figure 3:**
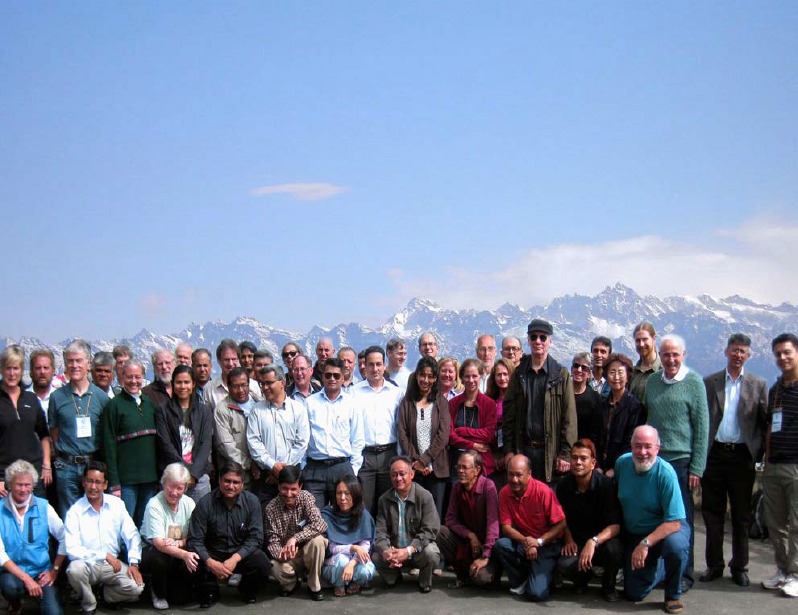
The International Advisory Board and local staff meeting in Nagarkot, Nepal in October 2011

### 4.3 Pedagogical Support

On the ground, tremendous support is still needed to get the day-to-day teaching done. While PAHS has developed its own educational experts, the faculty is small, experts in all subjects are not locally available, and development of best practices in clinical teaching and feedback is in progress. Thus far UAlberta has provided undergraduate teachers for cardiovascular, pediatric and nephrology problem-based learning blocks.

### 4.4 Faculty Development

University of Alberta faculty members have provided numerous faculty development sessions in medical education over the past four years spanning the gamut from how to provide effective classroom presentations to how to accomplish workplace assessment.

A larger project to coach faculty in the best practices of formative feedback, a keystone of competency-based education, is underway.

An international group of rural family physicians are investigating ways to support new community teaching physicians in Nepal with appropriate faculty development. Led by a physician from McMaster University, the group has extensive expertise in international family medicine and rural family medicine in North America and includes a member from the University of Alberta group.

In another case, UAlberta is exploring a mechanism to bring a PAHS/Patan Hospital faculty member over for fellowship education in nephrology – a current gap in the clinical service at Patan and teaching at PAHS.

An additional future project is to start discussions on creating local faculty development programs such as mentorship and peer-review which encourages reflective teaching practice.

### 4.5 Post-graduate Medical Education

One of the needs of PAHS is to eventually develop post-graduate programs for their graduates. Initially, PAHS students will graduate and enter rural practice. The goal is that some of them will return for post-graduate education, and the work to set up such programs has begun.

PAHS offers preferential selection of students who have completed rural service to provide additional motivation for students to serve in rural Nepal.

One of the UAlberta faculty members will soon be spending a 1-year sabbatical at PAHS to assist it for the planning and development of competency-based clinical postgraduate residency training programs in general and emergency medicine program in particular. This will coincide with his collaboration in the competency-based medical education model being evolved at PAHS.

**Figure 4:**
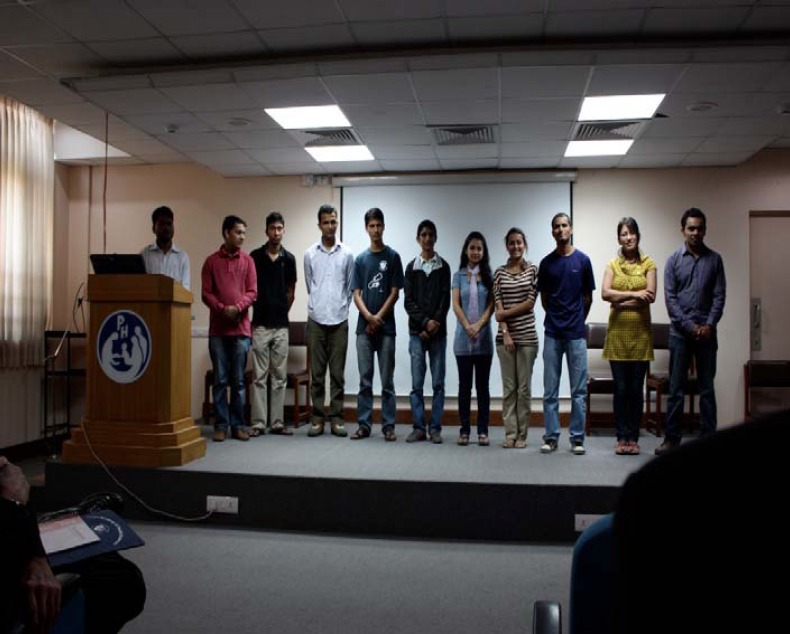
Medical students presenting in main PAHS auditorium

### 4.6 Fund Development

When Dr. David Cook, a renowned UAlberta medical educator and member of the PAHS IAB died in 2009, a legacy fund supporting medical education in Nepal was created. With the blessing of the University of Alberta Faculty of Medicine and Dentistry, and the assistance of a key fund developer, the David Cook Nepal Medical Education Fund serves as a mechanism for Canadians to make charitable contributions to support University of Alberta-PAHS initiatives, which still need funds to achieve their educational mission. UAlberta/FoMD fundraising staff are also keen to lend their expertise and advice to the PAHS International Advisory Board in an effort to build overall philanthropic support for PAHS.

### 4.7 Research Collaboration

Aligning with current work at PAHS, small amounts of Canadian-sourced funding have been obtained to research and implement elements of competency-based assessment and the corresponding faculty development.

Additionally, faculty members are part of a multi-centered research group on the effect international clinical rotations have on students and communities they visit. This Canadian project is in the planning phase. Last year the UAlberta sent its first medical students and residents to PAHS for an educational experience in global health.

### 4.8 Global Health Education

Educating University of Alberta students in international health at PAHS is a priority for the Office of Global Health. PAHS is a safe, supervised, robust environment for Canadian learners to engage directly in learning about developing nations and their health systems. 2012 will see the first students and residents from the UAlberta institution visiting PAHS. Care is being taken to ensure rigorous oversight over the process to avoid disrupting the learning of the PAHS students, and to enhance the UAlberta’s partnership with them.

This is not mere academic philanthropy. Faculty members take away tremendous inspiration, sense of value, collaborative projects and knowledge to apply back home. The value of this type of personal and professional development cannot be overstated.

### 4.8 Barriers in Supporting Medical Education Abroad

UAlberta faculty, students and staff engaged in the Nepal project understand many of the barriers to this style of international development work. Cultural barriers certainly exist; the largest of these are represented in the instability of the current political and funding system in Nepal, and a Canadian medical system that has moved away from the British-style medical hierarchy that exists in most Kathmandu teaching hospitals. And, of course, no UAlberta faculty have ever lived or practiced in the setting they are endeavoring to support.

Yet the challenges faced within the supporting group of such a medical school cannot be ignored. Faculty members are naturally inclined to pursue their own interests, which can lead to gaps in support, or overdevelopment in some areas. Drifting from the core mission is easy, and in this case could happen if the focus of the group moved from developing a rural medical school, to developing the services at the urban hospital it is housed at. On the other hand, for the effective retention of its graduates in the rural areas, PAHS has publicly committed to provide preferential enrollment in its postgraduate training program for those who will fulfill their mandatory rural service obligation. However, in order for PAHS to be accredited for launching the postgraduate residency training programs, it has no option other than to upgrade the Patan Hospital. Hence there is a need to strike a balance between its rural health mission and clinical excellence of its teaching hospital, The addition of students and residents, while meeting needs at the UAlberta, adds the complexity of integrating them into a resource-poor setting while providing mutual benefit.

**Figure 5:**
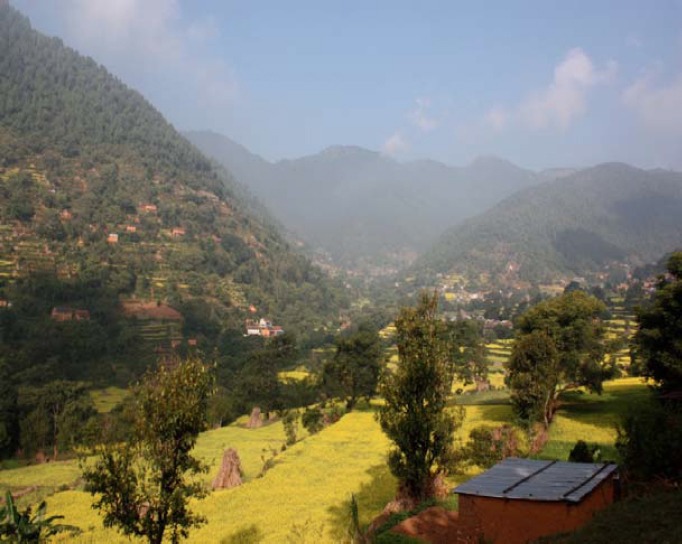
Typical landscape in rural Nepal

## 5. Discussion

The University of Alberta’s Faculty of Medicine and Dentistry has successfully evolved a functional support group that supports the mission of a novel medical school in a developing country. Much of the credit for success goes to the dedicated and visionary colleagues in Nepal, who have generously invited UAlberta into an effective collaboration. However, some of the success of the UAlberta group’s rapid evolution lies in its (at times inadvertent) use of several key principles:

1. Have a clear mission. The mission of this project is to enhance peace in Nepal by supporting rural-urban health equity. Clarity of that high level goal has engendered natural support from our members. The connection of routine daily work to the higher mission is clear and motivating.
2. Allow for organic growth. UAlberta has had a presence in Nepal, via Dr. Solez, for well over a decade. He knew the PAHS project was fertile ground for Canadian physicians, yet he and other Canadian leaders (Dr. Bob Woollard, UBC Chair of Family Medicine and the late Dr. David Cook, UAlberta 3M Teaching Fellow and international medical pedagogue) let the project attract the right people at the right time. Word-of-mouth, rather than targeted recruitment, has led to the natural selection of collaborators who are attracted to the mission. Their passion is key; all have a myriad of skills that can be brought to bear for the benefit of PAHS.
3. Leverage institutional support. But do not do so at the expense of the overall mission. In the span of four years, the group has expanded from the dedicated volunteerism of two faculty members to a recognized pillar of the Faculty of Medicine and Dentistry’s global health program involving fifteen faculty members. A yearly fundraiser has become a faculty-sponsored fund development project. Funding for research collaboration has been granted. UAlberta students and residents have been to PAHS, and an associate professor has been granted a one-year sabbatical to work with faculty at PAHS. Finally, as a marker of university-wide support, a memorandum of understanding has been drafted which will allow UAlberta faculty to engage in the project as faculty members, and not simply as volunteers. As support at the UAlberta was sought in all of these areas, none of it was felt to distract from the core mission – support rural health education in Nepal. The rewards for the University are those that come with effective global citizenship.

Eventually, the establishment of a formal link between a the University of Alberta and PAHS may be considered a ‘twinning’ relationship, as this multifaceted support is increasingly formalized and maintained (Macdonagh et al., 2002). Yet UAlberta has no special status on the IAB, and its representatives are considered equal among the myriad of international members coming from a dozen or more institutions worldwide. Each member has come to be on the board by virtue of their attraction to the mission. The advantage of this flexibly networked international advisory board is that the withdrawal of any one member does not threaten the integrity of PAHS. The limitations are that individuals may not be able to leverage all the resources of their home institution, and ongoing international collaboration can be at times slow and disorganized. (Oman et al. 2007)

The University of Alberta Nepal Group has modeled a coordinated, synergistic effort that has resulted in a rapid evolution of effective support for PAHS.

**Figure 6:**
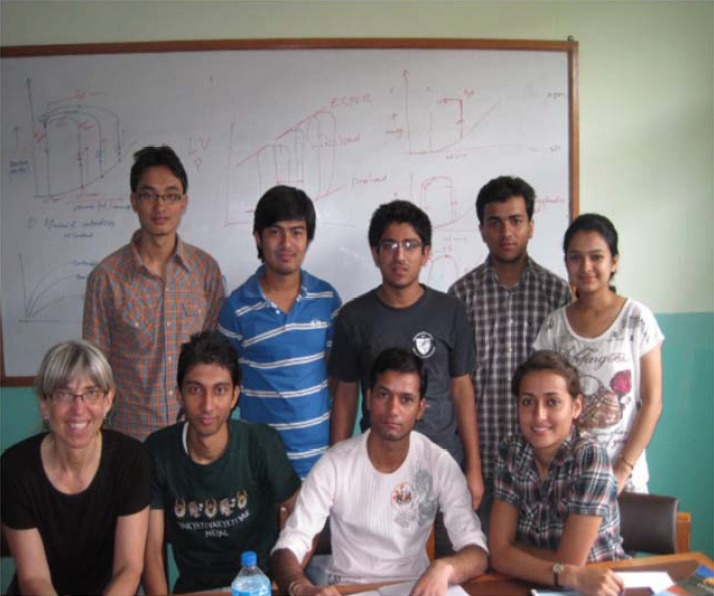
Dr. Bibiana Cujec teaching medical students at PAHS
